# What Do Real Alcohol Outpatients Expect about Alcohol Transdermal Sensors?

**DOI:** 10.3390/jcm8060795

**Published:** 2019-06-05

**Authors:** Pablo Barrio, Lidia Teixidor, Magalí Andreu, Antoni Gual

**Affiliations:** 1 Addictive Behaviors Unit, Clinical Neuroscience Institute, Clinic Hospital, 08036 Barcelona, Spain; lteixidor@clinic.cat (L.T.); magali221292@gmail.com (M.A.); tgual@clinic.cat (A.G.); 2 Department of Psychiatry and Clinical Psychobiology, University of Barcelona, 08036 Barcelona, Spain; 3 Grup de Recerca en Addiccions Clínic, Hospital Clínic de Barcelona, IDIBAPS, Universitat de Barcelona, Red de Trastornos adictivos (RETICS), 08036 Barcelona, Spain

**Keywords:** alcohol dependence, transdermal sensor, attitudes, stigma

## Abstract

Objective: Little is known about the potential acceptability of alcohol transdermal sensors among alcohol-dependent outpatients in routine clinical settings. The aim of the present study was to investigate patients’ attitudes towards alcohol transdermal sensors, as well as features associated with enhanced acceptability and usability. Methods: A cross-sectional survey among routine alcohol outpatients was conducted. The Drug Attitude Inventory (DAI-10) was adapted to the field of alcohol transdermal sensors for attitudes assessment. Likert-type and multiple-choice questions were used for acceptability and usability evaluation. Results: 68 patients completed the questionnaire, and the DAI-10 mean score was 3 (standard deviation (SD) = 6.5). Internal consistency revealed a Cronbach alpha of 0.613. The score of a single The score of a single Likert-type question about overall perceived value was 7.4 (SD = 2.6). Its correlation with mean DAI-10 scores was *r* = 0.633, with *p* < 0.001. Relapse prevention and a stricter treatment control from therapists were the main reported advantages. Perceived stigma was the main disadvantage. Features increasing device discretion would enhance its acceptability. Conclusions: The data suggest that transdermal sensors could play a role in the clinical treatment of alcohol outpatients and concerns regarding stigma should be taken into account. Future designs should try to minimize size and visibility and stigma concerns should be discussed with patients.

## 1. Introduction

Alcohol remains a first-order global health problem, with 15 million people affected in the European Union [1]. Recent publications in the United States also warn about an increase in the prevalence of alcohol use disorders during the last decade [2]. The impact it has on both individuals and society is of an enormous dimension, both medically and economically [3,4]. A significant share of these consequences is attributable to the severest form of alcohol use, i.e., alcohol dependence, which is currently considered a chronic disease, with a relapsing–remitting nature. Despite some controversies in the field, abstinence has been the prevailing therapeutic goal in most of the existing settings, being considered the safest and most efficient pathway to early recovery [5,6]. Therefore, for a great majority of professionals dealing with alcohol dependence, monitoring abstinence becomes an indispensable task. Traditional tools for abstinence assessment have consisted of patients’ self-reports and alcohol biomarkers. Despite recent improvements, especially in alcohol biomarkers [7,8], there remain some relevant limitations that could be overcome with the use of alcohol transdermal sensors.

Interestingly, in harm reduction or controlled drinking paradigms, alcohol transdermal sensors could also be of special utility, given the importance of quantifying the amount of alcohol ingested when patients chose to reduce their intake instead of abstaining. In quantifying the amount of alcohol consumed, transdermal sensors seem to be the most sensitive tool. In that sense, they could potentially be the ideal partner to the paradigm of heavy use over time [9], where the emphasis is put quantitatively on the amount of ethanol ingested rather than discretely qualifying patients as abstinent or not. This may suggest that transdermal sensors could be seen as a valuable tool to facilitate and improve digital phenotyping in addiction patients [10,11].

Transdermal sensors have been available for some years now. Their main use has been within the justice system [12]. There are also several studies in the literature assessing the devices’ validity and functioning, and there have already been randomized trials to evaluate their efficacy and patients’ experiences [13,14,15,16,17]. Together, they suggest that transdermal sensors could be effective in helping alcohol patients. They also point out towards good acceptability and feasibility. However, no study has been conducted in real practice, or everyday settings, so little is known about patients’ acceptability and attitudes towards such devices in the routine, daily clinical setting of patients undergoing routine treatment with no monetary compensation or legal imperatives. Since they offer an ongoing, 24 h measurement of alcohol through sweat, it could be hypothesized that patients could see them under an autonomy-restricting perspective. However, this has not been evaluated in a formal study. Therefore, it seems important, in order to better understand and anticipate patients’ acceptance toward these devices, to assess their attitudes towards alcohol transdermal sensors. Ideally, gathering end-users’ perspectives and preferences should facilitate device implementation in routine settings, and therefore enable maximum reach among real patients.

The aim of the present study, conducted among patients who have never used a transdermal device, was therefore two-fold: firstly, to investigate patients’ attitudes towards alcohol transdermal sensors, as well as their perceived utility; and secondly, to investigate what features of transdermal sensors would enhance patients’ acceptance and usability.

## 2. Methods

### 2.1. Study Design and Subjects

We performed a cross-sectional survey among alcohol-dependent patients attending the outpatient service of the Addictive Behaviors Unit of the Clinic Hospital of Barcelona. Subjects were specifically recruited among those attending the routine urine screening program. Ethics consent was granted from the Hospital Clinic Ethics Committee. Informed written consent was obtained from all participants prior to participation in the study.

### 2.2. Instrument

For this study, a specific questionnaire was designed and all questions were in Spanish. The questionnaire consisted of 3 main parts. The first was aimed at evaluating patients’ perceived utility and preferences regarding transdermal sensors. It consisted of 3 multiple choice and 7 Likert-type questions. The second part was designed to obtain patients’ beliefs and attitudes towards alcohol transdermal sensors as part of their treatment. Given the lack of similar research for this specific subject, we took advantage of the extensive literature regarding the Drug Attitude Inventory-10 [18], which was initially designed to test attitudes of patients with schizophrenia towards medication in order to correlate it to medication adherence. It consists of ten items; each one is scored with either 1 or −1, depending on whether the response signals a positive attitude towards medication or not. To avoid response bias, half of the items are worded positively, and half negatively. We therefore kept the same wording and the same order of the questions. Its score ranges from −10 to 10. We adapted its ten items to the present study, replacing the concept of medication by that of alcohol transdermal sensors. While such an adaptation of the Drug Attitude Inventory (DAI-10) has not been previously validated in the literature, two previous studies with alcohol patients (one cohort attending Alcoholics Anonymous groups and another cohort obtained from the same outpatient population of this study) performed a similar adaptation of the DAI-10, showing good psychometric properties [19,20]. In addition, we still considered it a good approach to capture patients’ attitudes, given the lack of more specific, validated instruments for our study aims. The third was devised to gather basic sociodemographic characteristics. At the beginning of the questionnaire, pictures of the existing transdermal devices were printed (the WristTAS and the SCRAM). Patients received no monetary compensation for participation in the study.

### 2.3. Procedure

The professional responsible for receiving patients and their urine specimens was an experienced nurse in the addiction field. Within a four-week period at the beginning of 2018, while the nurse received patients, patients were offered the option of participating in the questionnaire. To be included, patients had to be adults diagnosed with an alcohol use disorder and be attending the urine screening program. The main exclusion criteria were the presence of cognitive decline (either based on the nurse’s clinical judgment or patient history) and being in a state of intoxication. Patients with any other condition that, in the opinion of the investigators, could have compromised the validity of responses were not offered participation (e.g., an altered psychopathological state). Patients were reassured that all data provided would be kept totally anonymous. Once completed, questionnaires were kept safe until the end of the study, at which point they were analyzed.

### 2.4. Statistical Analysis

A descriptive analysis of sociodemographic data was conducted. The mean and the standard deviation were used for continuous variables; percentages were used for qualitative variables. Regarding the adapted version of the DAI-10, an internal consistency analysis was carried out with Cronbach’s alpha. Concurrent validity was assessed via bivariate correlations between the DAI-10 total score and a 0 to 10 Likert scale measure assessing the potential perceived value of alcohol transdermal sensors as part of patients’ treatment. Finally, in order to evaluate if attitudes were influenced by any specific variable, we conducted a lineal regression model with DAI-10 scores as the dependent variable and age, sex, level of instruction, therapeutic objective, and length of urine testing as predictors. Analyses were conducted with SPSS (IBM Corp. Released 2015. IBM SPSS Statistics for Windows, Version 23.0., IBM Corp, Armonk, NY, USA).

## 3. Results

During the study period, a total of 110 patients attended the urine screening program. Of that, 80 subjects were offered participation in the study (reasons for exclusion: intoxication of 11 patients, cognitive decline of eight patients, other reasons were not specified for 11 patients). A total of eight patients declined participation and four subjects accepted but did not return a questionnaire. That left a total of 68 patients completing the questionnaire. The mean age of the sample was 52.9 years (standard deviation (SD) 12.3). The 66% majority were men, and 39% of the sample were currently employed. Regarding education status, 14% had primary education, 28% had secondary education, 28% had a technical qualification, and 30% had a university qualification. Most of the patients (72%) underwent screening twice a week, 20% once a week, and 8.2% less than once a week. Most of the patients (78%) reported abstinence to be their therapeutic objective. Only a small minority (18%) reported drinking reduction as their aim. Patients had been attending the screening program for an average of 11 months (SD 10.5).

The main advantages and disadvantages attributed to transdermal sensors by patients and their frequencies can be seen in Table 1. Three patients spontaneously reported a decrease in human contact as a potential downside of transdermal sensors. Factors associated with enhanced acceptability and its rating from patients in a scale from 0 to 5 can be seen in Table 2. For both tables, a sex-specific analysis was also conducted. The main difference observed was for stigma, women presenting almost a 2-fold percentage of response compared to men.

**Figure 1:**
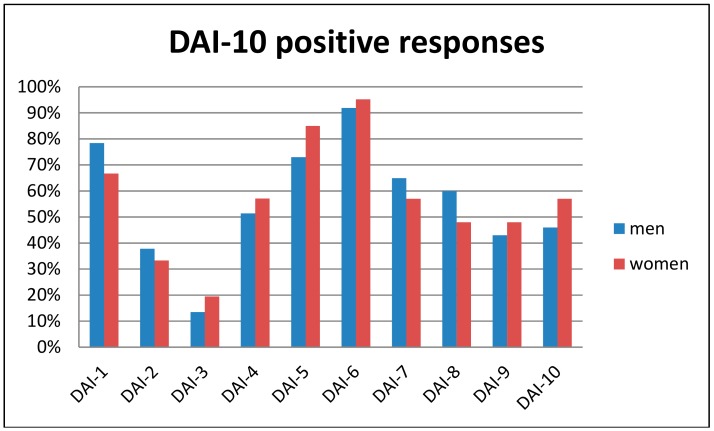
Percentage of positive responses to the Drug Attitude Inventory (DAI-10) questionnaire for each item and according to sex.

In a 0 to 10 scale, patients reported a mean score of 6.6 (SD 3.4) regarding the acceptance of a transdermal sensor if their therapist prescribed it. Regarding the adapted DAI-10 questionnaire, the mean score was 3.0 (SD 6.5). Internal consistency, measured with Cronbach’s alpha, was 0.613, indicating fair reliability. The question about the overall perceived value of alcohol transdermal sensors showed a mean of 7.4 (SD 2.6). The correlation between this and the DAI-10 score, as a means to investigate concurrent validity, was *r* = 0.633, with *p* < 0.001. The regression model revealed no significant predictor of DAI-10 scores. The differences according to sex regarding the percentage of positive responses to each DAI-10 item can be seen in Figure 1.

Table 3 reports preferences regarding different kinds of devices for transdermal sensors. As can be seen, almost all patients preferred a device similar to a nicotine patch (63%), while the rest of the options were far less frequently preferred.

## 4. Discussion

In this study, we aimed to investigate patients’ attitudes towards alcohol transdermal sensors. Globally, we believe the results suggest that patients perceive alcohol transdermal sensors as a potentially useful and valuable treatment device.

The adapted version of the DAI-10 scored above average among our sample. Similarly, the overall perceived value as rated in a 0 to 10 scale was high (7.4), and the correlation between both was rather high and clearly significant, suggesting a good concurrent validity. Additionally, the internal consistency of the adapted questionnaire, measured with Cronbach’s alpha, was fair. These results suggest the tentative conclusion that the results of the adapted DAI-10 questionnaire could be considered to be reasonably valid.

Looking at patients’ responses, the majority stated that preventing relapse and accomplishing their therapeutic goals regarding alcohol were the main functions of transdermal sensors. What is also interesting to note is the significant proportion of respondents that reported “showing professionals or their family that they do not drink” as a main function of transdermal sensors. It suggests that transdermal sensors could play an important role in patients’ interaction with both professionals and their social network.

In line with the most frequently reported inconvenience of transdermal sensors (i.e., stigma associated with wearing it), the features that could most enhance the device’s acceptability were precisely those that seemed to be designed to reduce the device’s visibility (small size and discreteness). Further supporting these findings, the most preferred physical device was a patch similar to the ones used for nicotine replacement therapy. In line with our findings, previous reports about transdermal sensors have also emphasized stigma and embarrassment as major concerns reported by patients [21,22]. What is also worth mentioning is that patients’ responses suggest that providing continuous feedback about the information gathered by the device is also essential in order to enhance patients’ acceptability and usability.

The results from the previous survey by Alessi and colleagues [17], which was conducted among patients who wore a real sensor, suggest that stigma and embarrassment were not major concerns. This is in sharp contrast to our results. The difference could be explained by the different sample selection and study design procedures, since all patients in the aforementioned survey were gathered from studies conducted with contingency management procedures, and the majority of subjects belonged to non-clinical samples, suggesting a potential selection bias. For example, in one of the studies patients were heavy drinkers not seeking treatment. That means that social desirability and the wish to abstain might have been quite different in both samples. Another potential explanation could be the fact that patients wore the SCRAM as a foot bracelet. This could be considered a discrete device and therefore stigma concerns would have been reduced.

Looking at patients’ responses, it seems that physical discomfort is also a main preoccupation among patients. It is probable that similar recommendations to the ones for dealing with stigma and embarrassment could be given. These were reduced size and visibility, which should also increase comfort with the device.

Overall, and in line with the increasing importance that patient-centered care is gaining in medical settings [23], we expect this study to offer relevant insights into patients’ perspectives, motives, and attitudes towards alcohol transdermal sensors. We expect knowledge about patients’ needs and worries to be increased, so that when, in the near future, they are offered to incorporate transdermal sensors into their treatment program, they can be offered this in a more realistic, friendly, and efficient manner.

Turning to the limitations of the present study, a key issue in interpreting all studies using questionnaires is social desirability [24]. Addiction itself is especially prone to such bias [25,26]. Therefore, although the questionnaires were completely anonymous, the presence of a social desirability bias cannot be totally ruled out.

Other relevant limitations should also be taken into account when interpreting our findings. First, we developed a new questionnaire. Although it was based on an extensively validated one (the DAI-10), it must be acknowledged that our study was not focused on questionnaire validation; therefore, more measurements could have been obtained in order to better validate it. That being said, the internal reliability and the concurrent validity were fair. Another important limitation stems from the fact that all patients belonged to a single outpatient center, a fact that might diminish external validity. Similarly, all patients were recruited from the urine screening program, so the question remains as to whether similar or different results would be observed in other clinical settings. It is also important to mention that the nature of this study was mainly descriptive. However, we tried to find some predictors of patients’ attitudes towards transdermal sensors, but found no significant predictors, a fact that could partly be due to insufficient statistical power. Also relevant is the fact that no systematic screening instrument for the detection of cognitive decline or other excluding conditions was used. The clinical nature of the study also precluded an extensive evaluation of patients’ clinical characteristics. Finally, four questionnaires were not fully completed and, therefore, we had a minor proportion of missing data, which we excluded from analysis.

## 5. Conclusions

Though our data suggest that concerns about stigma and embarrassment should be taken into account when planning the implementation of transdermal sensors in routine settings (more notably for female patients), our findings, together with those of previous studies, encourage further efforts to bring transdermal sensors to routine, clinical samples of alcohol-dependent patients.

Future designs of transdermal sensors should aim at reduced size and visibility, thus lowering stigma concerns. Patients should also be allowed full access to the information provided by the sensor.

## Figures and Tables

**Table 1:** Advantages and disadvantages attributed to transdermal sensors.

Advantages	Percentage of Responders (Both Sexes)	Male Responders	Female Responders	*p*-Value (Male vs. female)
To accomplish treatment goals	56%	54.8%	61.9%	0.415
To prevent relapse	41%	31%	57.1%	0.08
To show professionals I do not drink	28.8%	28.6%	28.8%	0.631
To show my family I do not drink	28.8%	23.8%	38.1%	0.700
To have stricter control from my therapists	41%	40.5%	42.9%	0.545
**Disadvantages**	**Percentage of Responders**			
Stigma associated with device	46%	36.6%	66.7%	0.049
Feeling in control all the time	23%	19.5%	24%	0.368
Physical discomfort	41.5%	39%	42.9%	0.550

**Table 2:** Features associated with enhanced acceptability (rated from 0 (lowest) to 5 (highest)).

Characteristics	Mean (SD) Both Sexes	Males	Females	*p*-Value (Male vs. female)
The sensor has a small size	4.2 (1.4)	4.1 (1.5)	4.3 (1.2)	0.627
The sensor is discrete	4.3 (1.4)	4.1 (1.5)	4.4 (1.1)	0.553
Information is never shared without my permission	2.1 (2.1)	1.9 (2.1)	2.5 (2)	0.984
Information is displayed in a web or app where I can always see it	4.1 (1.5)	3.9 (1.7)	4.4 (1.1)	0.920
I can erase the information I want whenever I want	2.0 (3.4)	2.1 (3.8)	1.8 (2.1)	0.725

**Table 3:** Preferred devices for transdermal sensor implementation.

Device	Percentage of Responders (Both Sexes)	Male Responders	Female Responders
Nicotine patch	63%	63%	62%
Subcutaneous implant	10%	12.5%	4.8%
Wrist-watch or similar device	13%	15%	9.5%
Dental implant	2%	2.5%	0%
